# Dental Infections and Systemic Disease—Treatment and Results*Read before the Section on Stomatology at the Sixty-Seventh Annual Session of the American Medical Association, Detroit, June, 1916. Printed in *Journal A. M. A.*, Sept. 16, 1916.

**Published:** 1916-10

**Authors:** Ernest E. Irons

**Affiliations:** Chicago


### DENTAL INFECTIONS AND SYSTEMIC DISEASE—TREATMENT AND RESULTS.*

BY ERNEST E. IRONS, M.D., CHICAGO.

From the medical standpoint as well as from the dental, the object of treatment of infections of the teeth is the conservation, as far as possible, of the structure and the function of the teeth, and the prevention of disabling systemic disease. So far as the physician is concerned the present discussion is in no sense a propaganda to prove

* Read before the Section on Stomatology at the Sixty-Seventh Annual Session of the American Medical Association, Detroit, June, 1916. Printed in *Journal A. M. A.*, Sept. 16, 1916.

anything with respect to the methods of treatment of teeth by the dental profession. It is rather a recital of facts which have come to light in the course of an effort on the part of the physician to understand the cause and mechanism of production of a large group of more or less disabling ailments which for years have baffled him in his efforts to cure them. It is true that there have developed many other angles to the question, but these latter have been largely secondary, and the main problem has been and is now that of relieving patients from their disabilities by as conservative and yet as effective methods as possible. Every new proposal or discovery in medicine, or in any field of human activity, passes through certain phases of reaction on the part of the professions concerned, characterized by incredulity, popularity, abuse, and finally, if found of value, takes its place among recognized procedures.

This has never been better illustrated than in the present discussion of alveolar abscess and its relation to systemic disease. It would appear that the great prevalence of alveolar abscess was until recent years as little suspected by many of the dental profession as by physicians, although the importance of oral sepsis was pointed out years ago by such pioneers as Miller and Hunter. Now that the facts are becoming known, in large part by reason of the perfecting of roentgenographic technic, many readjustments of practice, both dental and medical, are being found necessary. It is the difficulties of these readjustments and not the fault of the demonstration of abscesses, which have led to certain temporary misunderstandings, for the correction of which the only essential is a frank discussion, and then sympathetic team-work between dental surgeon and physician.

In this discussion of the medical aspects of dental infection I shall limit myself more particularly to forms of disease associated with alveolar abscess. The more superficial lesions, such as pyorrhea and gingivitis, probably exert an unfavorable influence on the general health, and conversely, systemic disturbances may furnish the conditions favorable to their development; but as a rule they do not appear to give rise to the metastatic lesions so commonly associated with alveolar abscess. Extensive pyorrhea may result in lateral alveolar abscesses, and possibly give rise to distant metastases, but a study of the medical complications associated with dental disease leads one to the view that infections about the teeth conform to the same principles as do surgical lesions elsewhere, and that it is the closed process which usually gives rise to general infection.

I suppose every dentist will agree that a definite and recognized alveolar abscess should be eradicated, whether it is causing general disease or not. Such a position is in keeping with the best tenets of American dentistry.

It must be recognized also that the final decision as to diagnosis and proposed treatment of a dental lesion should properly lie with the dental surgeon, to whose opinion the physician should defer; but in order that his opinion may be worth having, the dental surgeon must acquire an adequate knowledge of the pathology as well as the mechanical treatment of the dental structures. The sins of omission in this matter have not been confined to the dental profession, however, for physicians have been notoriously remiss in the examination of patients, and have overlooked many sources of disease, including those of the mouth, which now are clearly evident.

The observance of ordinary rules of professional courtesy by the physician, the dentist, and, I might add, the roentgenographer will go far toward making successful team-work possible, but each must make himself professionally worthy to deserve this courtesy, and must be willing to listen to the views of his colleagues.

Before passing to a consideration of the results of treatment from a medical standpoint, I wish to refer to certain misconceptions which have been introduced into the problem.

The startling frequency of alveolar abscess and other extensive dental infection has so impressed many physicians and not a few dentists that they are ready to ascribe all human ailments to disease of the teeth, and to attempt to cure them all by the extraction of teeth without definite proof of incurable dental disease, or even presumptive evidence of the causal relation of the dental infection to the systemic disease.

This extreme and wholly unwarranted position has brought about a reaction, and in some quarters the pendulum has oscillated to the other extreme, at which are found a group of protesters, who are ready to declare that diseases of the teeth have no relation to infections in other parts of the body, and who from their attitude would seem to assume that the presence or absence of alveolar suppurations is a matter of no concern either to dentist or physician. Now looking at the problem for the moment from an impersonal point of view, one can see that neither of these positions can be right, but that the truth lies somewhere between.

Another group of physicians and dentists, enthusiastic, somewhat less radical than those just mentioned, have assumed that in a patient suffering from evident disease, such as arthritis, in whom also an alveolar abscess is found, there is necessarily a direct etiologic relation between the two, and that drainage of the abscess will surely result in cure of the arthritis. It is true that occasionally this is the sequence of events, but it often happens that the alveolar abscess is only one of several areas of chronic infection in the body, and may indeed be co-ordinate with arthritis, both being secondary to some previously existing infection. The perspective of the enthusiast in any specialty is likely to be narrow, and he does not appreciate the importance of the field of his colleagues.

Examination of the blood to determine the presence of leukocytosis has been proposed as a guide to the dentist in determining whether or not a tooth showing doubtful roentgenographic findings should be extracted. The desire of dentists to study patients more thoroughly is laudable, but in this instance the presence or absence of leukocytosis can be of but little value unless all other causes of leukocytosis, such as infections in tonsils, sinuses, the genito-urinary and other organs of the body are first excluded, and since this necessitates a complete medical examination. which is not usually available, the test must therefore be far less reliable in routine dental work than the expert examination of the patient's mouth and roentgoenogram by the dentist.

The importance of alveolar abscess as a factor in the production of systemic disease has been already discussed by my predecessors in this symposium. Statistics on this subject have come largely from those who are interested in special lines of inquiry, and the relation of alveolar abscess to systemic disease has been determined by the study of groups of patients selected on account of the presence of some clinically striking lesion, such as arthritis.

An examination of the converse proposition is of some interest from the point of view both of etiology and of treatment. An unselected group of 329 patients in a medical ward in Cook County Hospital was studied and the incidence of all discoverable infectious processes determined and tabulated for each patient. The patients were then classified according to final diagnosis, and the percentage of each type of infection in each disease group calculated. It was possible to obtain roentgenographic studies, most of which were confirmed by consultation with dental surgeons, in 124 of the 329 patients. Careful tabulation showed that the patients submitted to roentgenographic examination were fairly representative of the entire group. In forty-four per cent. of the 124 patients alveolar abscesses were found. In the arthritic group seventy-six per cent. had alveolar abscesses; in the group of nephritis and cardiovascular disease forty-seven per cent.; other diseases, including pneumonias, respiratory, gastro-intestinal, twenty-three per cent., or less than one-third of the percentage in the arthritic group. Abnormalities in tonsils, as expressed by hypertrophy, were present in forty-five per cent. of the arthritic group, in twenty-four per cent. of the cardiovascular group, and in nineteen per cent. of the remainder. Other chronic infections, such as those of sinuses or genito-urinary tract, were found in twenty-one per cent. of the arthritic, thirteen per cent. cardio-vascular group, and eleven per cent. of the other diseases. Syphilis, either from the history, clinical evidence or Wassermann, was found in twenty-three per cent. of the arthritic group, thirty-nine per cent. of the cardiovascular group, and thirteen per cent. of other diseases.

This group of patients is not entirely comparable to a group coming from a higher economic station in life, but the figures serve to demonstrate the relation of the incidence of infections to other forms of disease within the group.

All arthritis or other metastatic and systemic disease is not due to alveolar abscess or to tonsillar infection, of course, but the preponderance of such lesions in this group suggests that these infections may play an important part in the production of chronic arthritis and similar ailments, and probably of certain types of cardiovascular disease, not only the valvular, but the arteriosclerotic, and also certain forms of nephritis, in which cold and exposure are only part of the determining causes.

In the arthritic group therapeutic measures directed to the removal of infectious processes have been followed by gratifying results, even in rather unfavorable material. Our readmissions for recurrences of arthritis, which in former years were frequent, some patients returning as many as four times in a season, have been relatively fewer, and usually of patients who for some reason or other could not be submitted to proper surgical attention.

In a series of 100 cases of iritis studied by Dr. G. V. I. Brown, we found alveolar abscesses and other closed infections, as revealed by roentgenograms and expert dental consultation, in thirty-six per cent. of forty-seven private patients, and forty-five per cent. of fifty-three dispensary patients, and in eighteen per cent. of the 100 patients the disease of the eye appeared to be associated with dental infection.

In estimating the results which may be expected from the correction of alveolar abscess in any given case it is evident that the presence and influence of other infectious foci must be considered, and neither too great credit for success nor too great censure for failure should be imposed on the dental surgeon who deals with only one of the possible factors in the larger general problem of recovery of the patient. Nor can we always promise prompt cure, even in those patients whose illness clearly originated in dental infection, for after the apparent immediate source of infection is removed, there often remain the mechanical injuries of joints and eyes, or other tissues may have become secondary foci of infection, and recovery is a question of measures which shall build up the resistance of the patient by improving his general nutrition.

### VACCINES.

The question of the use of vaccines has vexed the physician quite as much as it has the dentist. Active immunization by the injection of specific vaccines has proved of enormous value in the prevention of certain diseases, such as typhoid fever, but the results of the use of vaccines in the treatment of persons already suffering from infections have been in general decidedly disappointing.

There are certain more or less theoretical considerations in regard to the effect of an injection of vaccine on the balance of immunity between invading organism and host in a lesion such as alveolar abscess, but inasmuch as this balance may be deflected as often against as in favor of the host, the theoretical as well as the practical results of vaccine injections as now practiced indicate that their value is extremely doubtful, and that in no case should their use be allowed to replace or hinder the employment of recognized dental and surgical procedures.

Under the most favorable conditions a vaccine could be only an adjunct to other treatment, and in the types of disease we are now discussing, adequate surgical treatment by the dentist usually renders this adjunct superfluous so far as concerns the medical care of the patient. The dentist deals with special types of tissue, but allowing for peculiarities in structure and function of the tissues involved, the same principles of immunity and surgical procedure apply to infections about the teeth as to other organs of the body. When proper surgical measures are carried out by the dentist, the natural resistance of the tissues of the body, including those about the teeth, is usually sufficient to overcome the infecting organism. It would be foolish and futile to attempt to substitute the injection of vaccines for drainage and other well-established methods of treatment of an alveolar abscess; when proper dental procedures have been carried out, vaccines become unnecessary. The same statement would appear to apply to the treatment of pyorrhea, in which the practice of injecting vaccines, as advised by those commercially interested in their production and sale, has led to absurdities as discreditable to the skill of the dental profession as are similar practices so common in the past among physicians.

### SUMMARY.

The attitude of the physician in studying his patient should not be that of the prosecuting attorney bent on convicting the teeth of being the cause of all disease, nor should the dentist assume the brief of the defendant, ignoring the evidence of roentgenograms and systemic disease.

Both must work in harmony, each believing in the sincerity and good-will of the other, observing professional courtesy and laboring to place the patient in the best physical condition possible.

There seems to be no question that in many cases alveolar abscesses are the source from which invading organisms pass into the circulating blood and produce lesions in joints, eyes, nerves and other structures of the body. It is also true that alveolar abscesses, like tonsillar and other infection, may be latent so far as any marked effect on general health is concerned. Nevertheless, they are potential sources of trouble, and from the medical standpoint, and I believe from the dental, should be eliminated. The recognition of the important relation of alveolar abscess to systemic disease is a great step forward, and the period of extreme neglect of the past will never return. The readjustments in methods of diagnosis and in the establishment of a rational conservatism will come gradually as we learn to appreciate the interdependence of all organs of the body and the community of interest of the dental and medical professions. And this advance in turn calls for better medical education, better dental education.

The difficulties and misunderstandings from which we are now emerging afford one instance among many of the necessity of providing educational facilities which shall produce broad-minded professional men who can appreciate the problems of departments of human medicine other than their own.

